# Neuroimaging characteristics of dementia with Lewy bodies

**DOI:** 10.1186/alzrt248

**Published:** 2014-04-09

**Authors:** Elijah Mak, Li Su, Guy B Williams, John T O’Brien

**Affiliations:** 1 Department of Psychiatry, University of Cambridge School of Clinical Medicine, Box 189, Level E4 Cambridge Biomedical Campus, Cambridge CB2 0SP, UK; 2 Wolfson Brain Imaging Centre, Cambridge CB2 0QQ, UK

## Abstract

This review summarises the findings and applications from neuroimaging studies in dementia with Lewy bodies (DLB), highlighting key differences between DLB and other subtypes of dementia. We also discuss the increasingly important role of imaging biomarkers in differential diagnosis and outline promising areas for future research in DLB. DLB shares common clinical, neuropsychological and pathological features with Parkinson’s disease dementia and other dementia subtypes, such as Alzheimer’s disease. Despite the development of consensus diagnostic criteria, the sensitivity for differential diagnosis of DLB in clinical practice remains low and many DLB patients will be misdiagnosed. The importance of developing accurate imaging markers in dementia is highlighted by the potential for treatments targeting specific molecular abnormalities as well as the responsiveness to cholinesterase inhibitors and marked neuroleptic sensitivity of DLB. We review various brain imaging techniques that have been applied to investigate DLB, including the characteristic nigrostriatal degeneration in DLB using positron emission tomography (PET) and single-photon emission computed tomography (SPECT) tracers. Dopamine transporter loss has proven to reliably differentiate DLB from other dementias and has been incorporated into the revised clinical diagnostic criteria for DLB. To date, this remains the 'gold standard' for diagnostic imaging of DLB. Regional cerebral blood flow, 18 F-fluorodeoxygluclose-PET and SPECT have also identified marked deficits in the occipital regions with relative sparing of the medial temporal lobe when compared to Alzheimer’s disease. In addition, structural, diffusion, and functional magnetic resonance imaging techniques have shown alterations in structure, white matter integrity, and functional activity in DLB. We argue that the multimodal identification of DLB-specific biomarkers has the potential to improve ante-mortem diagnosis and contribute to our understanding of the pathological background of DLB and its progression.

## Introduction

Dementia with Lewy bodies (DLB) is the second most common form of neurodegenerative dementia following Alzheimer’s disease (AD), accounting for approximately 15% of cases at autopsy [1]. Characterised by cognitive fluctuations, visual hallucinations, and motor Parkinsonism, DLB shares both clinical and pathological features with other dementia types, including Parkinson’s disease dementia (PDD) and AD. Hence, DLB is a frequently misdiagnosed condition, and previously established consensus criteria are limited by low sensitivity. In light of this uncertainty in diagnosis, and with important implications for subsequent patient management, more reliable imaging markers are needed to help distinguish DLB from other subtypes of dementia. In this review, we provide a literature summary of the principal neuroimaging techniques used to investigate DLB in terms of its differentiation from other dementia types. The diagnostic standard for all mentioned studies is clinical criteria except where otherwise stated.

## Radionuclide imaging techniques

Nuclear imaging modalities such as single-photon emission computed tomography (SPECT) and positron emission tomography (PET) represent well established, reliable imaging methods to assess molecular changes in DLB (Table 1).

**Table 1:** Summary findings in DLB compared to AD, PDD and HC

**Comparisons with DLB**	**Structural imaging: MRI and DTI**	**Molecular and functional imaging: SPECT, PET, and fMRI**	**Metabolic differences: MRS**
**AD**	Relatively preserved MTL volume in DLB compared to AD	Significant reduction in FP-CIT binding in caudate and putamen compared to AD	Normal levels of NAA/Cr and myo-inositol levels in DLB compared to AD
	`Smaller substantia innominata and putamen in DLB compared to AD	Hypometabolism in occipital cortex and visual association, preservation of posterior cingulate	
	A greater posterior predominance of FA change in DLB as opposed to a more diffuse pattern of change in AD	Lower levels of amyloid deposition compared to AD	
	Reduced FA in the pons and the left thalamus in DLB compared to AD	DLB patients show increased connectivity of the DMN in DLB compared to AD	
		Greater activation of the superior temporal sulcus in DLB compared to AD during a fMRI motor task	
**PDD**	More pronounced atrophy in DLB in the temporal, occipital and parietal lobes	Increased amyloid deposition in DLB compared to PDD	
**HC**	Reduced GM in temporal, parietal, occipital, and subcortical structures in DLB when compared to HC	DLB show significant reduction in FP-CIT binding in caudate and putamen compared to HC	Reduced white matter NAA/Cr in DLB compared to HC
			Increased Cho/Cr ratios in DLB compared to HC
	Reduced FA in parieto-occipital white matter tracts in DLB compared to HC	Hypometabolism in occipital cortex in DLB compared to HC	
		Compared to HC, DLB patients show preservation of functional activity associated with lower visual areas	
		Decreased V5/MT functional activity in response to motion stimuli is found in DLB compared to HC	

AD, Alzheimer’s disease; Cho, choline; Cr, creatine; DLB, dementia with Lewy bodies; DMN, default mode network; DTI, diffusion tensor imaging; FA, fractional anisotropy; fMRI, functional magnetic resonance imaging; GM, gray matter; HC, healthy controls; MRI, magnetic resonance imaging; MRS, magnetic resonance spectroscopy; MT, middle temporal; MTL, medial temporal lobe; NAA, N-acetylaspartate; PDD, Parkinson’s disease dementia; PET, positron emission tomography; SPECT, single-photon emission computed tomography.

### Single-photon emission computed tomography

The focus of SPECT on DLB has been on the demonstration of alterations in the dopamine transporter (DAT), reflecting changes in the nigrostriatal pathway, and the analyses of cerebral perfusion and metabolism.

#### Dopamine transporter loss

**Figure 1:**
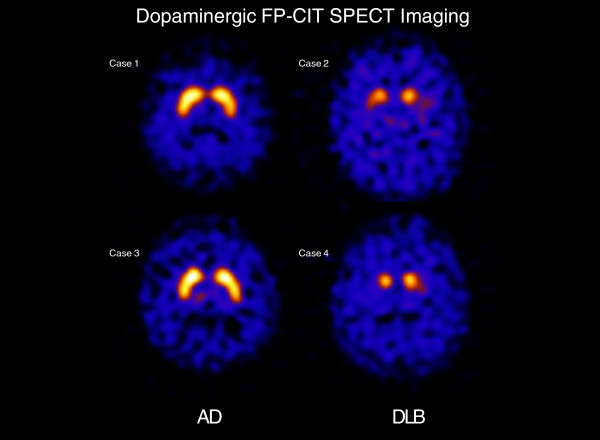
**Comparison of FP-CIT scans between Alzheimer’s disease (AD) and dementia with Lewy bodies (DLB).** In the DLB group, reduced uptake is evident in the caudate while more extensive loss is found in the putamen.

Imaging ligands, such as ^123^FP-CIT, have been developed for SPECT to visualise DAT loss *in vivo*. Reduced binding in the striatum reflects dysfunction or loss of nerve terminals in the substantia nigra. Previous autopsy literature has established that nigrostriatal degeneration and subsequent DAT loss is far more severe in DLB compared to AD [2]. In healthy volunteers and patients with AD, the ligand is taken up in the caudate and putamen where neurons expressing the DAT are concentrated. In DLB, however, ligand uptake is almost absent in the putamen, and is reduced in the caudate (Figure 1). Consistent with an earlier single-site study [3], a phase III multicentre imaging study demonstrated a sensitivity and specificity of 78% and 90%, respectively, to distinguish DLB from AD [4]. The effectiveness of ^123^FP-CIT-SPECT in the distinction between DLB and AD is further confirmed in another autopsy study (88% sensitivity and 100% specificity) [5].

DAT loss is related to the clinical feature of motor parkinsonism though, as DAT loss can be demonstrated before clinical parkinsonism is apparent, it may also be present in those without motor features. In addition, one study reported decreased DAT levels to be associated with visual hallucinations in DLB [6], suggesting the possible involvement of mesocortical dopaminergic pathways in the clinical phenotype of DLB. Recognition of the diagnostic value of dopaminergic SPECT in DLB is reflected in its incorporation in the revised consensus criteria for DLB, which allow for the diagnosis of probable DLB with evidence of a low DAT uptake in SPECT or PET imaging in the presence of one (rather than the usual two) core feature of DLB [7].

#### Perfusion studies

Several radiopharmaceuticals are available for regional cerebral blood flow SPECT: ^99m^Tc hexamethylpropyleneamineoxime, N-isopropyl-p-[^123^I]iodoamphetamine and ^99m^Tc-ethyl cysteinate dimer. The majority of studies have consistently highlighted a distinctive pattern of occipital hypoperfusion in DLB when compared to AD, affecting both primary visual cortex and visual association areas, and including the precuneus [8-10]. Lobotesis and colleagues [8] reported that blood perfusion in DLB and AD differed only in occipital regions and distinguished DLB from AD and from control subjects with 65% sensitivity and 87% specificity. Another SPECT study using N-isopropyl-p-[^123^I]iodoamphetamine also demonstrated relatively preserved medial temporal lobe perfusion in DLB in addition to occipital hypoperfusion in DLB when compared with AD [11]. In an earlier study by Colloby and colleagues [12], a region of interest (ROI) analysis revealed decreased cerebral blood flow in AD compared to DLB in the medial temporal lobe, bilateral striatum and in the right thalamus. More recently, the same group used a spatial covariance analysis approach and concluded that multivariate analysis of blood flow SPECT data showed good diagnostic accuracy for distinguishing DLB from AD [13].

In summary, the two different SPECT methods (DAT and perfusion) are both useful in diagnosing DLB, but DAT imaging appears to be more robust and accurate compared to perfusion [14]. On the contrary, SPECT was unable to discriminate between DLB and PDD, thus supporting the concept that these two forms of dementia belong to the same continuum spectrum, but are distinct diseases from AD [15].

### Positron emission tomography

PET also allows molecular investigation of dementia subtypes but is less widely available and more expensive than SPECT. In addition, the versatility of PET imaging is due to the variety of different radioisotopes available, allowing investigation of different functional systems; for example, energy metabolism with 18 F-fluorodeoxygluclose (FDG), cholinergic pathways with N-11C-methyl-4-piperidyl acetate, the dopaminergic system with 18-fluorodopa and other ligands for the vesicular monoaminergic transporter, and cerebral amyloid deposition with Pittsburgh compound B and^18^F labelled compounds, such as 18flurodopa, 18 F Flutemetamol and 18 F Flubetapir.

#### Metabolism

Consistent with SPECT findings of occipital hypoperfusion in DLB patients, FDG-PET studies have established a distinctive pattern of hypometabolism in the occipital cortex [16,17] and visual association cortices, with relative preservation of the posterior cingulate, which is normally markedly affected in AD (the so-called 'cingulate island' sign). Furthermore, occipital hypometabolism has been linked by some to visual hallucinations in DLB [17]. Using 18fluorodopa, Klein and colleagues [16] did not reveal any differences in dopaminergic deficit profiles between DLB and PDD.

#### Amyloid deposition

Most of the research done with amyloid PET imaging has been focused on AD, which is consistently associated with elevated levels of uptake, especially in the prefrontal cortex, midline and lateral parietal cortex, temporal cortex and striatum. Although the pathological hallmarks of DLB are Lewy bodies and Lewy neurites, cortical amyloid-beta deposition is often seen. The importance of amyloid-beta burden in DLB and cognitive impairment remains to be elucidated. Amyloid imaging studies in DLB have yielded variable results. While some studies have reported similar amyloid-beta deposition in DLB and AD, most studies report lower mean cortical amyloid-beta ligand binding in DLB patients, with both AD and DLB patients showing elevated amyloid burden compared to their healthy controls [18].

Differences in cortical amyloid burden between DLB and PDD have been investigated in recent amyloid PET studies. Edison and colleagues [19] demonstrated that cortical amyloid load is significantly raised in over 80% of DLB patients, while amyloid pathology is infrequent in PDD. This finding is largely in accordance with the literature reporting elevated cortical amyloid deposition in DLB compared to PDD [18], which is aligned with the presence of greater cortical Alzheimer pathology in DLB [20]. Clinically, it is also noteworthy that increased amyloid deposition has also been found to be associated with a more aggressive rate of cognitive deterioration and visuospatial impairments in DLB [21,22]. Future research clarifying the influence of amyloid deposition might be important for informing treatment decisions with the potential availability of effective anti-amyloid agents.

#### Cholinergic pathways

Autopsy findings of profound cholinergic deficits in DLB (greater than in AD) have led to the application of PET ligands to evaluate the cholinergic system in DLB *in vivo*, such as N-[11C]methylpiperidin-4-yl acetate and propionate. Klein and colleagues [16] have demonstrated widespread reductions of acetylcholinesterase activity in DLB, particularly affecting the posterior regions, while similar reductions have also been reported in the thalamus [23]. This pattern of cholinergic deficiency is comparable to that seen in Parkinson’s disease [24,25]. On the other hand, choline acetyltransferase activity, particularly in the neocortex, is markedly reduced in DLB compared to AD, consistent with the autopsy findings [26]. The spatial distribution of cholinergic deficiencies also appears to be different from AD, where deficits are most prominent in the temporal lobes while the thalamus is relatively spared [23].

#### Nigrostriatal projections

PET studies using ^11^C-dihydrotetrabenazine have also examined nigrostriatal projections in DLB and AD, and previous studies have demonstrated its potential to differentiate DLB from AD. Compared to controls, significantly decreased binding of ^11^C-dihydrotetrabenazine was predominantly found in the posterior and anterior putamen and caudate nucleus in DLB, while differences were found between AD and controls [27].

## Structural magnetic resonance imaging

Besides nuclear imaging methods, the clinical utility of structural neuroimaging with magnetic resonance imaging (MRI) for differential diagnosis of dementias is also well established. Structural MRI has been widely used to compare regional structural changes in patients with DLB against AD, PDD and their healthy controls. In this section, we summarise the principal findings using MRI to distinguish DLB from other subtypes of dementia (Table 1).

### Comparison between dementia with Lewy bodies and Alzheimer’s disease

**Figure 2:**
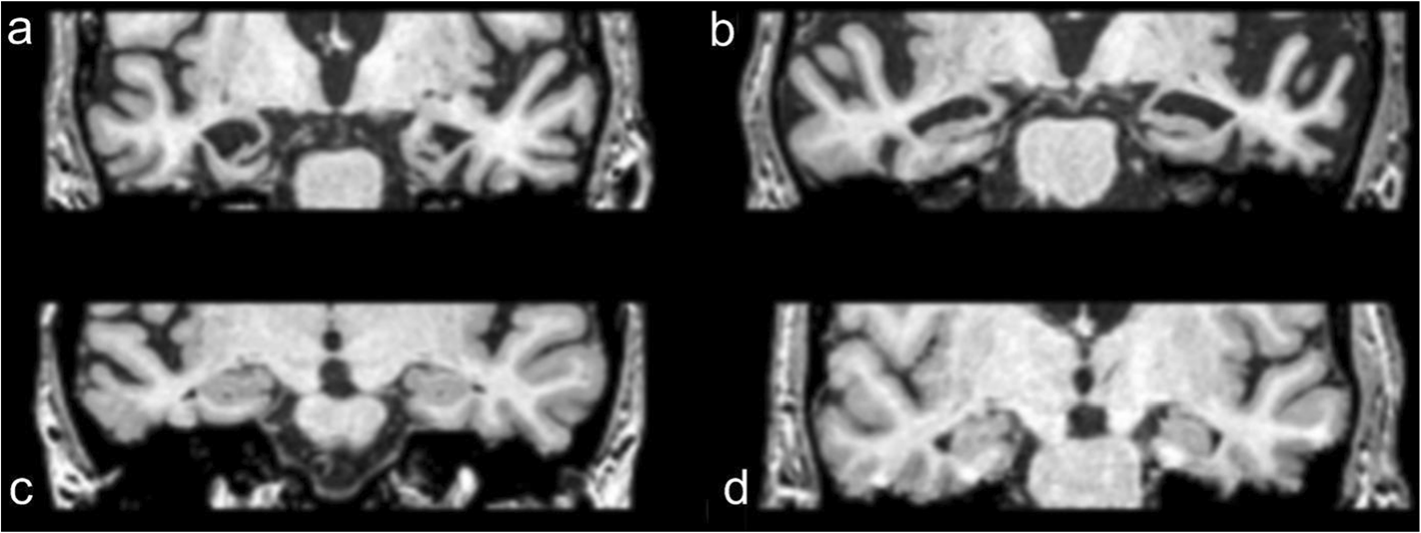
**Preservation of medial temporal lobe in dementia with Lewy bodies compared to Alzheimer’s disease. (a)** A 76-year-old female with Alzheimer’s disease (AD). **(b)** A 72-year-old male with AD. **(c)** A 75-year-old female with AD. **(d)** A 73 year old male with dementia with Lewy bodies.

Both cross-sectional and longitudinal studies have shown that DLB is associated with less pronounced global atrophy than AD [28]. The most consistent finding is the relative preservation of the medial temporal lobe (MTL) in DLB compared to AD [28] (Figure 2). Burton and colleagues [29] demonstrated the clinical significance of MTL atrophy for distinguishing DLB from AD in pathologically confirmed cases, although another autopsy study argued that the presence of MTL atrophy may not rule out a diagnosis of DLB, especially amongst patients in the oldest old-age category (>85 years) [30]. In addition, Burton and colleagues [29] reported a strong correlation between hippocampal atrophy and amyloid-beta plaques and neurofibrillary tangles but not Lewy body-associated neuronal inclusions, suggesting that gray matter (GM) loss in DLB could be due to concomitant AD pathology. Hippocampal atrophy in DLB and AD has also been investigated, showing less severe atrophy in DLB than in AD when compared to healthy controls [31]. Similarly, other studies have demonstrated that the entorhinal cortex, CA1, and subiculum areas of the hippocampus may be most affected in AD compared to DLB [28,32].

In parallel with the MTL findings, patients with DLB generally demonstrate relative preservation of episodic memory capabilities compared to AD while experiencing impairments in attentional and visuospatial tasks. Hence, Ballmaier and colleagues [33] hypothesised that frontal lobe structures could be spared in DLB and the reciprocal connections with relatively preserved temporal regions would be affected to a lesser extent too. Although their study revealed greater frontal GM loss in AD relative to DLB [33], other studies have not found differences between these patient groups [34].

Conversely, the midbrain appears to be more affected in DLB than in AD, with the substantia innominata showing greater atrophy [35,36]. The substantia innominata contains the nucleus basalis of Meynert, which is highly involved in the cholinergic neurotransmitter system. In addition, Whitwell and colleagues [35] demonstrated that increased dorsal mesopontine GM atrophy distinguished patients with clinically diagnosed DLB and AD, and this finding was further confirmed in an autopsy study involving patients with high likelihood DLB [37]. These findings are thus in agreement with greater cholinergic dysfunction in DLB, and perhaps relate to the presence of midbrain synuclein pathology (see the 'Cholinergic pathways' section).

In light of the ^123^FP-CIT SPECT functional changes involving the loss of DAT in DLB [4], associated subcortical structures have also been investigated relative to AD. Cousins and colleagues [38] have observed reduced putamen volume in patients with DLB, which may be associated with the striatal synuclein pathology.

### Comparison between dementia with Lewy bodies and Parkinson’s disease dementia

There have also been attempts to compare GM loss between DLB and PDD. These results have converged to reveal a pattern of more pronounced GM loss in DLB compared to PDD. This is in agreement with the PET and pathological findings of greater amyloid burden in DLB. It is noteworthy that localisations of GM reductions in DLB relative to PDD appear to be variable amongst studies. While Burton and colleagues [39] did not detect different cortical atrophy profiles between DLB and PDD, a voxel-based morphometry (VBM) study by Beyer and colleagues [40] of PDD, DLB, AD, and healthy controls revealed GM reductions in the temporal, parietal and occipital lobes in DLB. Besides the temporal and parietal areas, Lee and colleagues [41] also identified striatal and occipital GM reductions. A different pattern of structural and functional correlations between DLB and PDD was recently revealed [42]. In particular, decreased GM volume in associative areas, namely the left precuneus and inferior frontal lobe, correlated with visual hallucinations in DLB but not in PDD patients. The variation in patient population, sample size and subtle differences in imaging analysis methodology may account for such mixed effects in comparing GM reduction between the DLB and PDD groups; for example, VBM has been shown to be highly sensitive to co-registration and normalisation errors.

### Cortical thickness analyses

Recent advances in image processing allow automatic extraction of whole brain cortical thickness information from structural MRI. Cortical thickness has been shown to demonstrate high precision and sensitivity in detecting alterations in morphology resulting from neuropathological changes. Hence, it has been used in increasing numbers of studies as a marker to separate AD and PDD from healthy controls. Recently, a multivariate classification study of cortical thickness demonstrated 82% sensitivity and 85% specificity for differentiating AD from DLB [43]. Specifically, AD was characterised by regional thinning of the parahippocampal, subgenual cingulate regions and temporal pole, whereas cortical thinning in DLB was localised in the middle and posterior cingulate, superior temporo-occipital and lateral ortibofrontal regions. It is interesting to note that the greater temporal involvement in AD compared to DLB has been one of the most consistent findings on structural imaging whether on visual inspection, ROI or VBM studies [33,44].

### White matter hyperintensities

White matter hyperintensities (WMHs) are areas of high signal intensity commonly detected using T2-weighted or fluid inverted recovery sequences in periventricular and deep subcortical white matter regions. In older people they are often a marker of cerebral small vessel disease, accumulating over time. WMHs are commonly attributed with loss of myelin and axons, and mild gliosis [3], but can also reflect amyloid angiopathy and other pathologies. Over the past decade, visual rating scales and volumetric assessments have been developed to quantify WMH load. Although the clinical significance of WMHs in dementia remains to be determined, several studies have found WMHs to be associated with cognitive impairments. A longitudinal study comparing progression of WMHs in AD, DLB and PDD revealed a greater burden at baseline of WMHs in AD compared to healthy controls, DLB and PDD, with no subsequent differences in the rate of progression between groups [45]. The importance of WMHs in DLB remains poorly understood. Thus, future research is needed to investigate region-specific WMHs and examine potential differences in functional correlations between dementia subtypes.

## Functional MRI

Active task and resting state functional MRI (fMRI) are the main neuroscience tools to examine cerebral function related to cognitive tasks or during rest through changes in blood oxygen level-dependent signal. There are still relatively few fMRI studies in DLB, but different patterns of functional connectivity between AD and DLB have been reported (Table 1). A recent resting-state fMRI study showed both increased connectivity between the precuneus and regions in the dorsal attention networks and decreased connectivity with prefrontal and visual cortices in DLB compared to the AD group [46]. Another data-driven independent component analysis study demonstrated increased connectivity in the default mode network in DLB compared to AD [47]. This finding contrasts with reported connectivity dysfunctions between the posterior and anterior portions of the default mode network in AD [48]. Kenny and colleagues [49] found greater connectivity between the putamen and frontal, temporal and parietal regions in DLB patients compared with AD patients, and argued that this might be associated with Parkinsonian features in DLB. Consistent with relative preservation of memory function in DLB compared with AD, hippocampal connectivity was not found to be different in DLB compared to healthy controls. Conversely, the left hippocampal connectivity was greater in AD compared with controls, which might reflect potential compensatory mechanisms.

Given the high prevalence of visuoperceptual impairments in DLB patients, task-based fMRI studies have investigated the functional integrity of the visual system in DLB. A task-based fMRI study involving visual presentations of colour, face and motion paradigms found greater activation in the superior temporal sulcus in DLB compared to AD during the motor part of the tasks [50]. Another task-based fMRI study did not find any significant differences in functional response between DLB and healthy controls to checkerboard, objects or motion stimuli in V1 and V2/V3, suggesting a relative preservation of function in lower visual areas. Interestingly, ROI analysis revealed decreased V5/MT (middle temporal) activation in response to motion stimuli in the DLB group [51]. Whether these abnormalities at higher levels of the visual system contribute towards the characteristic visuoperceptual impairment and visual hallucinations needs further empirical evidence. In summary, these results seem to indicate that functional abnormalities in DLB affect the visual association areas rather than the primary visual cortex.

## Diffusion tensor imaging

Diffusion tensor imaging (DTI) provides *in vivo* information about white matter microstructural integrity by utilising the anisotropic nature of diffusion in neuronal white matter tracts [52]. White matter diffusion characteristics are commonly assessed by means of mean diffusivity (MD) and fractional anisotropy (FA). MD increases with the degeneration of structural barriers that normally restrict the Brownian motion of water molecules, and reductions in FA occur as diffusion consequently becomes less directionally oriented.

DTI studies in AD have consistently found elevated MD in the hippocampus and decreased FA in the main limbic pathways. Much less is known about DTI changes in DLB. Previous DTI studies on DLB used ROI-based or voxel-based techniques with considerable variability in their findings. Some studies have reported diffusion abnormalities of the corpus callosum and the frontal, parietal, occipital, and, to a lesser extent, temporal white matter when compared to controls, while other studies have found very little change in DTI parameters compared to controls and AD patients [53,54]. The modest involvement of the temporal lobe is consistent with the relative preservation of global neuropsychological measures and memory domain in DLB compared to AD. Two DTI studies in DLB patients have also identified white matter alterations in the longitudinal fasciculus [54,55]. Considering the significant role of longitudinal fasciculus in ventral visual pathway, these abnormalities could be associated with visuospatial impairment and visual hallucinations in DLB patients. In addition, elevated MD in the amygdala was also found in DLB, which was associated with Unified Parkinson’s Disease Rating Scale scores [54]. However, AD was not accompanied by loss of GM in this brain region, implying a different pathologic mechanism, such as vacuolisation. However, no changes in diffusivity measures were identified between DLB and AD [54]. On the contrary, a recent DTI study demonstrated distinct patterns of white matter alterations between DLB and AD, with a more focal posterior predominance of FA change in DLB (parieto-occipital) as opposed to a more diffuse pattern of change in AD. DLB was also associated with reduced FA in the pons and the left thalamus compared to AD [56].

## Magnetic resonance spectroscopy

1H magnetic resonance spectroscopy (MRS) measures the peak signals from several different metabolites within a single examination period, and it has increasingly been used in differential diagnosis of dementia through the identification of respective spectroscopic profiles of various dementia subtypes (Table 1). In AD, the metabolite N-acetylaspartate is consistently found to be reduced in temporal lobe tissue (approximately 15%) [57], and is associated with disease severity [58]. In addition, increases in myo-inositol (15%) are also commonly reported [57]. In comparison, DLB is characterised by relatively normal N-acetylaspartate/creatine and myo-inositol levels, suggesting neuronal integrity and a lack of gliosis, respectively [59]. Further research with larger sample sizes will determine the clinical utility of these findings in distinguishing DLB from other types of dementia.

## Data analyses

At present, neuroimaging findings in dementia are generally derived from group-level analyses. While these have enriched our understanding about the neurobiological differences between DLB and other dementia types, the diagnostic value of most neuroimaging methods is still limited due to the lack of sensitivity and specificity when applied at the individual subject level. As such, there has been increased emphasis on maximizing the utility of more advanced data analysis methods to bridge the gap between the basic research and clinical practice. In this regard, novel machine learning techniques have been developed to allow individual classification of patients. Machine learning techniques extract features from neuroimaging data and construct models of different dementia types and healthy population. These models have been used to differentiate AD from controls, mild cognitive impairment, and frontotemporal lobar degeneration [60], while the potential of this approach in classification among dementia types has been highlighted by a recent autopsy-based machine-learning study by Vemuri and colleagues [61] involving pathologically confirmed dementia patients (AD, DLB, and frontotemporal lobar degeneration). Besides the advent of machine learning techniques, multimodal neuroimaging studies have become increasingly popular as researchers are recognising the benefits from integrating more than one imaging modality (for example, jointly analysing brain volume with perfusion or white matter changes). Recent multimodality strategies have shown superior predictive power than using any single modality domain in the diagnosis of AD [62-64], and distinguishing AD from frontotemporal lobar degeneration [65] and mild cognitive impairment [66]. Similar approaches have recently been applied also to the differentiation of DLB from other conditions. Goto and colleagues [67] integrated MRI striatal volumetric data with occipital perfusion SPECT to distinguish patients with mild DLB from patients with mild AD with high sensitivity and specificity. Kantarci and colleagues [68] achieved increased accuracy (98%) for distinguishing DLB from AD by combining information from occipital FDG uptake, global Pittsburgh compound B retention, and hippocampal volume. Given the multifactorial nature for pathological involvement in patients with DLB, differentiation between DLB and other dementia types will be aided by combining imaging modalities that are sensitive to different components of the disease process.

Longitudinal analyses of neuroimaging data will also provide information about the trajectory of the disease and its underlying neurobiological changes. By allowing the assessment of brain changes over time in individual subjects using multiple serial MRI scans, longitudinal designs have the advantage of reducing within-subject variation. Different rates of cerebral atrophy in dementia types could also inform differential diagnosis. In fact, DLB patients showed a much slower rate of global atrophy (0.4%) compared to AD patients (1.1%) [69].

## Conclusions

This review summarises the current imaging literature of DLB in the context of its differentiation from other causes of dementia, discusses the increasingly important role of imaging biomarkers in differential diagnosis, and outlines promising areas for future research. Collectively, the imaging findings have yielded important insights into the underlying pathophysiology of this condition while showing potential promise in improving clinical differentiation of DLB from other types of dementia.

To date, the marked reduction of dopaminergic activity in the basal ganglia is the most characteristic imaging finding of DLB, and has been considered as a significant step in aiding the clinical diagnosis of DLB. Cerebral perfusion studies have also revealed a distinctive pattern of deficits in parietal and occipital regions. At the structural level, another robust observation concerns the relative preservation of MTL in DLB that is consistent with preserved memory functions. Other techniques such as DTI, MRS, and fMRI coupled with novel analytical approaches have also revealed information for differential diagnosis of DLB.

While the field has made substantial progress in delineating the imaging characteristics associated with dementia subtypes, the ability to detect structural patterns that enable accurate prediction of diagnosis for specific individuals ultimately determines the clinical value of MRI and the measurements obtained from it. The reliable application of these methods in routine radiological practice may be facilitated by non-expert-dependent, automated methods of analysis.

In terms of practical considerations, SPECT and PET are both generally well tolerated investigations with few contra-indications, though both involve radiation exposure, limiting the number that any one subject should have. In most countries, SPECT is more widely available than PET. MRI is now widely accessible but more unsuitable for those with claustrophobia, pacemakers or metallic implants in or around the head. fMRI requires specialist stimulus presentation, which limits its applicability to research studies. In addition, some computerized paradigms in task-based fMRI might not be suitable for elderly dementia patients. Other modalities, particularly DTI and MRS, require extensive post-processing analysis time, also potentially limiting their routine application for clinical use.

It is worth mentioning the limitations of the current research. At present, the overwhelming majority of neuroimaging studies in DLB are cross-sectional, relatively small in size, and in participants in established stages of the disease. Therefore, larger prospective longitudinal studies are warranted to confirm the utility of many imaging techniques and monitor disease progression in early disease stages as well as at risk individuals and patients with mild cognitive impairment. Furthermore, studies involving multimodal neuroimaging data and larger cohorts are likely to make novel contributions in evaluating the utility of combined biomarkers in DLB.

## Note

This article is part of a series on *Lewy Body Dementia*, edited by Ian McKeith and James Galvin. Other articles in this series can be found at http://alzres.com/series/LewyBodyDementia.

## Abbreviations

AD: Alzheimer’s disease; DAT: Dopamine transporter; DLB: Dementia with Lewy bodies; DTI: Diffusion tensor imaging; FA: Fractional anisotropy; FDG: 18 F-fluorodeoxygluclose; fMRI: Functional magnetic resonance imaging; GM: Gray matter; MD: Mean diffusivity; MRI: Magnetic resonance imaging; MRS: Magnetic resonance spectroscopy; MTL: Medial temporal lobe; PDD: Parkinson’s disease dementia; PET: Positron emission tomography; ROI: Region of interest; SPECT: Single-photon emission computed tomography; VBM: Voxel-based morphometry; WMH: White matter hyperintensity.

## Competing interests

EM, LS and GBW have no competing interests. JTO’B has acted as a consultant for GE Healthcare and Lilly.
